# A phase III, randomized, two-armed, double-blind, parallel, active controlled, and non-inferiority clinical trial to compare efficacy and safety of biosimilar adalimumab (CinnoRA®) to the reference product (Humira®) in patients with active rheumatoid arthritis

**DOI:** 10.1186/s13075-017-1371-4

**Published:** 2017-07-20

**Authors:** Ahmadreza Jamshidi, Farhad Gharibdoost, Mahdi Vojdanian, Soosan G. Soroosh, Mohsen Soroush, Arman Ahmadzadeh, Mohammad Ali Nazarinia, Mohammad Mousavi, Hadi Karimzadeh, Mohammad Reza Shakibi, Zahra Rezaieyazdi, Maryam Sahebari, Asghar Hajiabbasi, Ali Asghar Ebrahimi, Najmeh Mahjourian, Amin Mohammadinejad Rashti

**Affiliations:** 1 Rheumatology Research Center, Tehran University of Medical Sciences, Tehran, Iran; 2 AJA university of Medical Sciences Rheumatology research center, Tehran, Iran; 3 AJA university of Medical Sciences Internal medicine, Rheumatology Section, Tehran, Iran; 4 Department of Rheumatology, Loghman e Hakim Hospital, Shahid Beheshti University of Medical Sciences, Tehran, Iran; 5 Shiraz Geriatric Research Center, Shiraz University of Medical Sciences, Shiraz, IR Iran; 6 Department of Rheumatology, School of Medicine, Shahrekord University of Medical Sciences, Shahrekord AND Behcet’s Unit, Rheumatology Research Center, Tehran University of Medical Sciences, Tehran, Iran; 7 Department of Rheumatology, Al-Zahra Hospital, Isfahan, Iran; 8 Endocrinology and Metabolism Research Center, Institute of Basic and Clinical Physiology Sciences, Kerman University of Medical Sciences, Kerman, Iran; 9 Rheumatic Diseases Research Center, Faculty of Medicine, Mashhad University of medical Sciences, Mashhad, Iran; 10 Guilan Rheumatology Research Center, Department of Rheumatology, Razi Hospital, School of Medicine, Guilan University of Medical Sciences, Rasht, IR Iran; 11 Tabriz University of Medical Sciences, Connective Tissue Reserch Center, Tabriz, Iran; 12 Tehran University of Medical Sciences, Tehran, Iran; 13 Tehran University of Medical Sciences, Faculty of Pharmacy, Tehran, Iran

**Keywords:** Adalimumab, Biosimilar, CinnoRA®, Rheumatoid arthritis

## Abstract

**Background:**

This study aimed to compare efficacy and safety of test-adalimumab (CinnoRA®, CinnaGen, Iran) to the innovator product (Humira®, AbbVie, USA) in adult patients with active rheumatoid arthritis (RA).

**Methods:**

In this randomized, double-blind, active-controlled, non-inferiority trial, a total of 136 patients with active RA were randomized to receive 40 mg subcutaneous injections of either CinnoRA® or Humira® every other week, while receiving methotrexate (15 mg/week), folic acid (1 mg/day), and prednisolone (7.5 mg/day) over a period of 24 weeks. Physical examinations, vital sign evaluations, and laboratory tests were conducted in patients at baseline and at 12-week and 24-week visits. The primary endpoint in this study was the proportion of patients achieving moderate and good disease activity score in 28 joints-erythrocyte sedimentation rate (DAS28-ESR)-based European League Against Rheumatism (EULAR) response. The secondary endpoints were the proportion of patients achieving American College of Rheumatology (ACR) criteria for 20% (ACR20), 50% (ACR50), and 70% (ACR70) responses along with the disability index of health assessment questionnaire (HAQ), and safety.

**Results:**

Patients who were randomized to CinnoRA® or Humira® arms had comparable demographic information, laboratory results, and disease characteristics at baseline. The proportion of patients achieving good and moderate EULAR responses in the CinnoRA® group was non-inferior to the Humira® group at 12 and 24 weeks based on both intention-to-treat (ITT) and per-protocol (PP) populations (all *p* values >0.05). No significant difference was noted in the proportion of patients attaining ACR20, ACR50, and ACR70 responses in the CinnoRA® and Humira® groups (all *p* values >0.05). Further, the difference in HAQ scores and safety outcome measures between treatment arms was not statistically significant.

**Conclusion:**

CinnoRA® was shown to be non-inferior to Humira® in terms of efficacy at week 24 with a comparable safety profile to the reference product.

**Trial registration:**

IRCT.ir, IRCT2015030321315N1. Registered on 5 April 2015.

## Background

Rheumatoid arthritis (RA) is a chronic, inflammatory, autoimmune disease of unknown pathophysiology leading to peripheral and symmetrical joint synovitis. The primary systemic manifestations are pain, morning stiffness, fatigue, and weight loss [1–3]. In progressive forms, it may lead to cartilage damage, joint destruction, and joint swelling resulting in impaired physical function and premature morbidity [4–6]. RA mostly develops in the fourth and fifth decades of life, with 80% of the cases occurring between 35 and 50 years of age. The worldwide prevalence of RA is about 0.5–1.0% with a female/male ratio of 2.5:1.0 [7, 8]. Although the presence of chronic inflammation has been proposed as a contributing factor, the exact mechanism of developing RA is still unknown [9].

The management of RA aims primarily at improving patients’ quality of life (QoL), achieving low disease activity based on American College of Rheumatology (ACR) and European League Against Rheumatism (EULAR) criteria, and ultimately remission [1, 10]. The treatment options for RA include non-steroidal anti-inflammatory drugs (NSAIDs), glucocorticoids, conventional synthetic disease-modifying antirheumatic drugs (sDMARDs) and biological DMARDs (bDMARDs). RA treatment has developed considerably in recent years, with the early use of methotrexate (MTX) and the addition of targeted bDMARDs in patients with an inadequate response to MTX [10, 11]. In fact, concomitant use of bDMARDs and MTX has been associated with the greatest clinical outcomes in trials and has been approved as the standard of care for patients with moderate-to-severe disease [12]. The stage and severity of the joint condition, the balance between possible adverse effects and expected benefits, and patients’ preferences are amongst the influential factors in choosing a DMARD. MTX is the most frequently administered sDMARD and is used either as monotherapy or in combination with other anti-rheumatic drugs. The early onset of action and superior efficacy makes MTX the synthetic agent of choice in the treatment of RA [5, 13]. Similarly, biological agents such as anti-tumor necrosis factor-α (anti-TNF-α) monoclonal antibodies are effective in suppressing disease activity, inhibiting structural deterioration and maintaining physical function. Adalimumab, a fully humanized immunoglobulin (IgG1) monoclonal antibody is produced in genetically modified Chinese hamster ovary cells (CHO). Adalimumab consists of two identical heavy and two identical light chains that bind specifically to the transmembrane TNF, thus blocking the interaction of TNF-α with its receptor [4, 14–16]. Adalimumab was first approved by the US Food and Drug Administration (FDA) in December 2002 for the treatment of RA and is currently approved for the following indications: RA, juvenile idiopathic arthritis (JIA), psoriatic arthritis (PsA), ankylosing spondylitis (AS), Crohn’s disease (CD), pediatric CD, ulcerative colitis (UC), psoriasis (PsO), pediatric plaque PsO, hidradenitis suppurativa (HS), and non-infectious uveitis [17, 18].

Biosimilars are biotherapeutic products that are similar to the licensed biological reference products in terms of quality, efficacy, and safety, but often are provided at a lower price [19, 20]. CinnoRA® was developed by CinnaGen Company (Alborz, Iran) as a biosimilar to the innovator adalimumab product (Humira®). This study aimed to evaluate the non-inferiority of test-adalimumab (CinnoRA®) to the reference product in terms of efficacy, tolerability, and safety in patients with active RA.

## Methods

### Study design

In this randomized, double-blind, non-inferiority trial, a total of 136 patients with active RA were randomized in a 1:1 ratio to receive 40-mg subcutaneous injections of either biosimilar adalimumab (CinnoRA®, CinnaGen Co., Iran) or the reference product (Humira®, AbbVie Inc., USA) every other week along with methotrexate (15 mg/week), folic acid (1 mg/day), and prednisolone (7.5 mg/day) over a period of 24 weeks. The study was conducted in accordance with the principles of good clinical practice (GCP) and the declaration of Helsinki across 10 referral hospitals in Iran. All the procedures were approved by the Institutional Review Board (IRB) and Ethics committees of each hospital. Patients provided written informed consent forms before initiation of any study-related procedure. The trial is registered in the Iranian registry of clinical trials with the following identification code IRCT2015030321315N1.

Patients’ demographic information was recorded at baseline and a thorough medical examination was performed. Vital signs and laboratory examinations were taken from the patients at baseline and at the 12-week and 24-week visits. All the injections were administered by trained nurses at each study site. After initial screening and assessing eligibility, patients were randomized by permuted balanced block randomization with a block size of four. The randomization was implemented using telephone randomization by an independent contract research organization. Allocated treatments were administered to patients based on their enrollment number. Patients, nurses, and physicians were unaware of the size of the blocks and the allocated treatments, and the blinding was maintained till the end of the intervention. Information relating to demographic characteristics, contact history of subjects diagnosed with active tuberculosis (TB), medical history, and prior medications were collected. Body weight and height were measured and a complete physical examination was performed. Also, vital signs including heart rate and blood pressure were measured. Laboratory examinations, including hematological and blood chemistry assessment, measurement of rheumatoid factor, C-reactive protein (CRP), hepatitis B surface antigen, and hepatitis C antibody, urinalysis, and a pregnancy test in women, were performed during a fasting state.

### Participants

Adult subjects of each gender who met the following criteria were included in this study: age between 18 and 75 years; active RA diagnosed by EULAR criteria [21]; moderate-to-severe RA for at least 6 months; lack of response to conventional non-biologic anti-rheumatic drugs after at least 12 months of therapy; and the ability to read, understand, and sign the written informed consent form.

Patients with any of the following criteria were excluded from the study: active or latent TB with a purified protein derivative (PPD) tuberculin test more than 5 mm in size or abnormal chest X-ray (CXR); previous treatment with TNF inhibitors; known hypersensitivity to human immunoglobulin proteins or other components of adalimumab formulation; in women, pregnancy, current nursing, or intention to become pregnant during the study; positive serology test for hepatitis B or C or human immunodeficiency virus antibody; ACR functional class IV or wheelchair/bed bound; taking intravenous antibiotics during the 8 weeks prior to screening or receiving oral antibiotic treatment during the 2 weeks before screening; history of serious, relapsing, or chronic infection; hemoglobin less than 8.5 g/dl; platelet count less than 125,000/μl; leukocyte count less than 3500/μl; serum creatinine more than 2 mg/dl; concomitant use of NSAIDs or more than 10 mg/day of prednisolone; receiving intravenous, intramuscular, intra-articular or oral corticosteroids (prednisolone, more than 7.5 mg/day) in the previous 4 weeks; previous treatment with rituximab, azathioprine, or 6-mercaptopurine (6-MP); history of chronic heart failure (CHF); history of myocardial infarction (MI) or unstable angina pectoris within 12 months prior to screening; history of demyelinating diseases or multiple sclerosis; history of malignancy within 5 years prior to screening; and participation in the study judged by the physician to be potentially harmful to the patient.

### Efficacy and safety assessment

The percent of patients achieving good and moderate disease activity score (DAS)-based EULAR response was the primary endpoint of this study. The proportion of patients reaching ACR criteria for 20% (ACR20), 50% (ACR50), and 70% (ACR70) improvements after 24 weeks of treatment with adalimumab along with the disability index of the health assessment questionnaire (HAQ), and safety were the secondary endpoints of this study [22, 23]. The incidence of adverse events at each visit was recorded based on patients’ reports, vital signs, physical examinations, and laboratory tests.

### Statistical analysis

In a study conducted by Broeder et al., 89% of the patients who received adalimumab achieved EULAR response [24]. The sample size of 64 people in each group was estimated by a non-inferiority margin of δ = −0.18 that was based on clinical judgement with 90% power and a 0.025 one-sided significance level. Primary efficacy measures were evaluated using both intention-to-treat (ITT) and per-protocol (PP) populations, while ITT population was used for safety assessment. Data were analyzed using Student's independent samples *t* test, the Mann-Whitney U test, the normal approximation test, Pearson’s chi-square test, and Fisher's exact test. *P* values <0.05 were considered statistically significant. Data were analyzed using Stata 11.2 software (College Station, TX, USA) and plotted using GraphPad prism version 6.0 (GraphPad Software, USA).

## Results

**Fig. 1:**
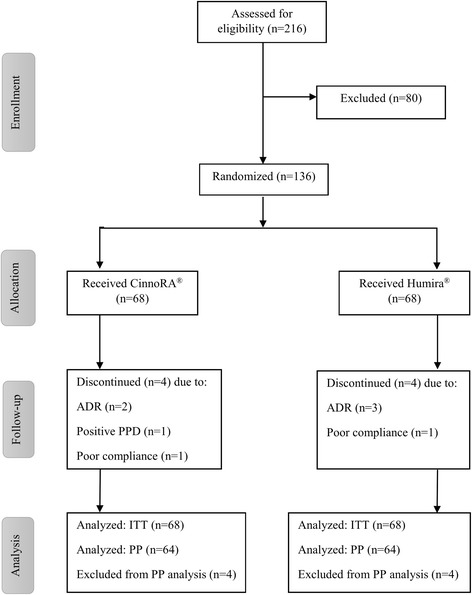
Trial profile. *ITT* intention-to-treat, *PP* per-protocol

A total of 216 subjects were screened across 10 hospitals in Iran, of whom 136 patients were considered eligible for participation in this study. Patients were enrolled in the CinnoRA® or Humira® arms (68 subjects in each arm) and 64 patients in each group completed the 24-week study period. Four patients in the CinnoRA® group withdrew from the study for the following reasons: adverse drug reactions (ADR, *n* = 2), positive PPD test (*n* = 1), and poor compliance (*n* = 1). Similarly, four patients in the Humira® group left the trial because of ADR (*n* = 3) and poor compliance (*n* = 1). The study profile is shown in Fig. 1.

Patients who were randomized to the CinnoRA® or Humira® arms had comparable baseline characteristics. The mean age of the participants in the CinnoRA® and Humira® groups was 48.29 ± 12.72 and 47.59 ± 11.48 years, respectively. The mean DAS in 28 joints based on erythrocyte sedimentation rate (DAS28-ESR) was 5.51 ± 1.24 in the CinnoRA® arm and 5.47 ± 1.28 in the Humira® arm. The baseline characteristics are summarized in Table 1.

**Table 1:** Summary of the baseline characteristics of the patients

Variable	CinnoRA®	Humira®	*P* value
Age	48.29 ± 12.72	47.59 ± 11.48	0.73
Sex, *n* (%)			
Male	10 (14.71%)	8 (11.76%)	0.85*
Female	58 (85.29%)	60 (88.24%)	
Swollen joint count, 28 joints	9.96 ± 7.39	9.46 ± 6.98	0.69
Tender joint count, 28 joints	9.46 ± 8.23	9.66 ± 7.97	0.88
Patient assessment of pain	67.21 ± 23.51	70.22 ± 21.93	0.44
Patient global assessment of disease activity	70.15 ± 20.35	70.74 ± 22.21	0.87
Physician’s global assessment of disease activity	68.97 ± 17.38	70.44 ± 17.68	0.63
CRP (mg/L)	21.40 ± 25.98	18.90 ± 23.90	0.57
ESR (mm/h)	32.65 ± 21.24	31.12 ± 24.01	0.69
HAQ	1.25 (1.38)	1.38 (1.13)	0.56^†^
DAS28-ESR	5.51 ± 1.24	5.47 ± 1.28	0.87
RF	63.76 ± 57.22	76.95 ± 65.66	0.22

Data are shown as mean ± SD and were analyzed using the independent *t* test unless stated otherwise. *CRP* C-reactive protein, *DAS28* disease activity score in 28 joints, *ESR* erythrocyte sedimentation rate, *HAQ* health assessment questionnaire, *RF* rheumatoid factor. *Data were analyzed using Pearson's chi-squared test. ^†^Scores are shown as median (interquartile range) and were analyzed using the Mann-Whitney U test

At week 12, the DAS28-ESR values were 2.95 ± 1.30, and 2.96 ± 1.41 in the CinnoRA® and Humira® groups, respectively (*P* value = 0.97); at week 24 the DAS28-ESR values were 2.58 ± 1.06 in the CinnoRA® arm vs. 2.55 ± 1.14 in the Humira® group (*p* value = 0.88).

**Fig. 2:**
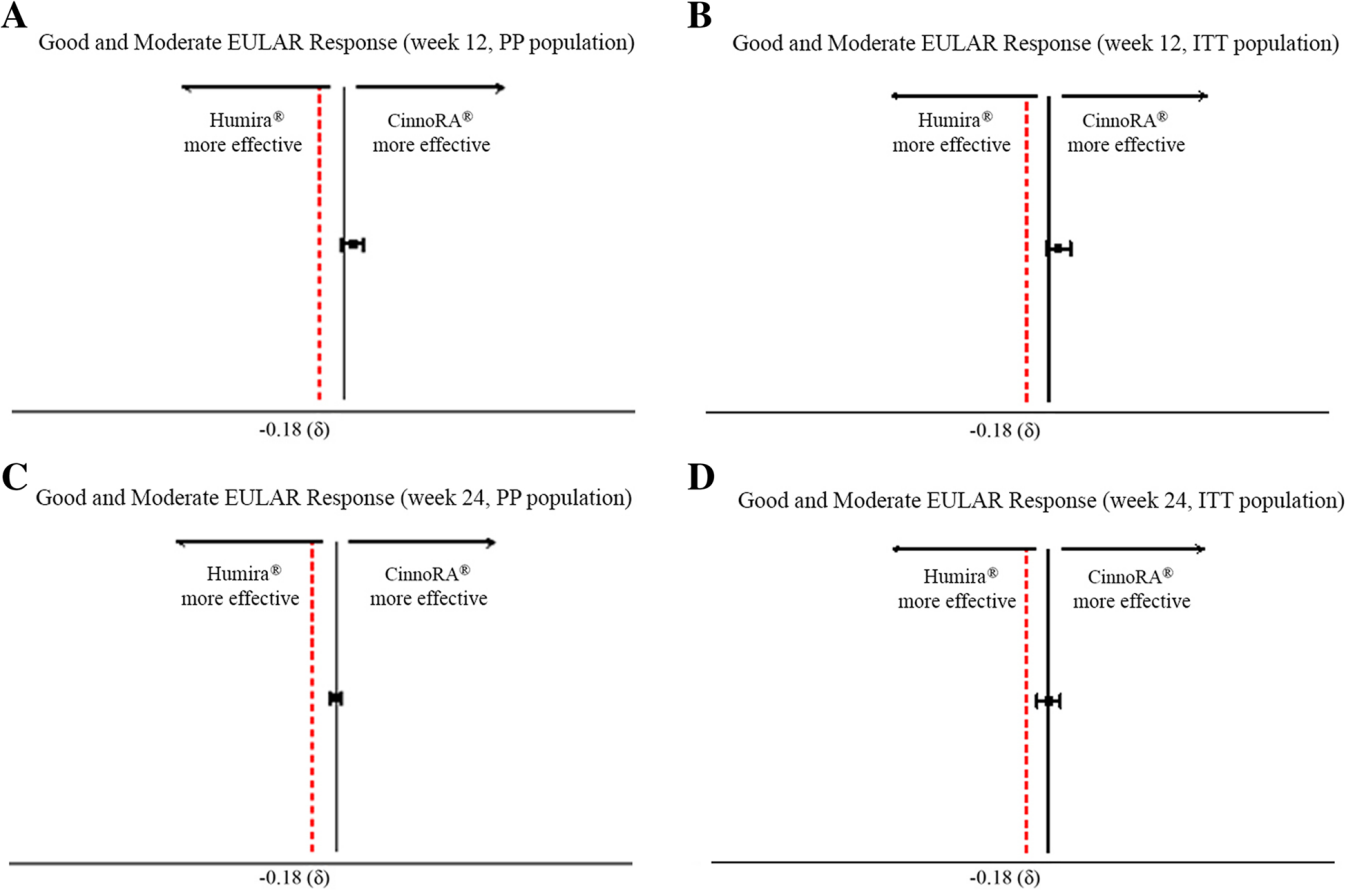
Evaluation of non-inferiority of test-adalimumab to reference adalimumab in terms of the proportion of patients who met good and moderate disease activity score in 28 joints based on erythrocyte sedimentation rate (DAS28-ESR) European League Against Rheumatism (*EULAR*) response in the per-protocol (*PP*) population at 12 weeks, 95% CI for the difference −0.009 to 0.16 (**a**); the intention-to-treat (*ITT*) population at 12 weeks, 95% CI for the difference −0.02 to 0.17 (**b**); the PP population at 24 weeks, 95% CI for the difference −0.04 to 0.04 (**c**); and the ITT population at 24 weeks, 95% CI for the difference −0.09 to 0.09 (**d**)

According to the PP population, the proportion of patients fulfilling moderate and good EULAR response criteria based on the DAS28-ESR at week 12 was 42.42% and 54.55% in the CinnoRA® arm compared to 37.88% and 51.52% in the Humira® group, respectively (cumulative 12-week good and moderate EULAR response in the PP population 97% in the CinnoRA® vs. 89% in the Humira® arm; *p* value = 0.08; CI for the difference = −0.9 to 16). At 24 weeks, 28.13% and 70.31% in the CinnoRA® vs. 31.25% and 67.19% in the Humira® arm met the criteria for moderate and good EULAR response, respectively (cumulative 24-week good and moderate EULAR response in the PP population 98% in the CinnoRA® vs. 98% in the Humira® arm; *p* value = 1; CI for the difference = −4 to 4). Similarly, in the ITT population, 41.18% and 36.73% achieved moderate EULAR response at 12 weeks in the CinnoRA® and Humira® arms, respectively, and 52.94% in the CinnoRA® arm and 50.00% in the Humira® arm achieved good EULAR response at 12 weeks (cumulative 12-week good and moderate EULAR response in the ITT population 94% in the CinnoRA® vs. 87% in the Humira® arm; *p* value = 0.14; CI for the difference = −2 to 17). At 24 weeks, moderate EULAR response was attained in 26.47% of patients in the CinnoRA® arm compared to 29.41% in the Humira® arm. However, good EULAR response was achieved in 66.18% of patients in the CinnoRA® arm and 63.24% in the Humira® arm (cumulative 24-week good and moderate EULAR response in the ITT population 93% in the CinnoRA® vs. 93% in the Humira® arm; *p* value = 1; CI for the difference = −9 to 9). Based on the prespecified margin of 20%, non-inferiority of test-adalimumab to the reference product in terms of the proportion of patients achieving good or moderate EULAR response was confirmed at both the 12-week and 24-week time points (Fig. 2, Table 2).

**Table 2:** Summary of DAS28-ESR, HAQ, and EULAR response measures

Variable		Week	CinnoRA®	Humira®	*P* value
DAS28-ESR		12	2.95 ± 1.30	2.96 ± 1.41	0.97*
		24	2.58 ± 1.06	2.55 ± 1.14	0.88*
HAQ		12	0.25 (0.88)	0.38 (0.88)	0.87**
		24	0.25 (0.63)	0.19 (0.63)	0.48**
EULAR response (PP)	No response, *n* (%)	12	2 (3.03)	7 (10.61)	0.28***
	Moderate response, *n* (%)		28 (42.42)	25 (37.88)	
	Good response, *n* (%)		36 (54.55)	34 (51.52)	
	No response, *n* (%)	24	1 (1.56)	1 (1.56)	0.92***
	Moderate response, *n* (%)		18 (28.13)	20 (31.25)	
	Good response, *n* (%)		45 (70.31)	43 (67.19)	
EULAR response (ITT)	No response, *n* (%)	12	4 (5.88)	9 (13.24)	0.37***
	Moderate response, *n* (%)		28 (41.18)	25 (36.76)	
	Good response, *n* (%)		36 (52.94)	34 (50.00)	
	No response, *n* (%)	24	5 (7.35)	5 (7.35)	0.96***
	Moderate response, *n* (%)		18 (26.47)	20 (29.41)	
	Good response, *n* (%)		45 (66.18)	43 (63.24)	

*DAS28-ESR* disease activity score in 28 joints based on erythrocyte sedimentation rate, *HAQ* health assessment questionnaire, *EULAR* European League Against Rheumatism, *PP* per-protocol, *ITT* intention-to-treat. *Data are shown as mean ± SD and were analyzed using the independent samples *t* test. **Scores are shown as median (interquartile range) and were analyzed using the Mann-Whitney U test. ***Data were analyzed using Fisher's exact test

The median (IQR) HAQ scores at 12 and 24 weeks were 0.25 (0.88) and 0.25 (0.63) in the CinnoRA® arm vs. 0.38 (0.88) and 0.19 (0.63) in the Humira® arm, respectively. The difference between treatment arms was not statistically significant at the respective time points (12 weeks, *p* value = 0.87; 24 weeks, *p* value = 0.48).

**Fig. 3:**
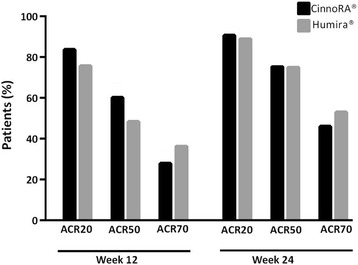
The proportion of patients achieving American College of Rheumatology 20%, 50% or 70% response (*ACR20*, *ACR50*, or *ACR70*) following treatment with test or reference adalimumab at 12 and 24 weeks

The proportion of patients achieving ACR20, ACR50, and ACR70 responses at the 12-week time point were 85%, 61%, and 28% in the CinnoRA® arm compared to respective values of 76%, 48%, and 36% in the Humira® arm. At week 24, 92%, 77%, and 47% of the patients in the CinnoRA® arm achieved ACR20, ACR50, and ACR70 responses, respectively, which was similar to those observed in the Humira® arm (89%, 75%, and 53%, respectively). No statistically significant difference was observed between treatment arms at either the 12-week or the 24-week time point (all *p* values >0.05, Fig. 3).

The incidence of adverse effects was comparable between patients who received test-adalimumab compared to those who took the reference product. Overall, a total of 24 patients (35.29%) in the CinnoRA® arm vs. 30 (44.12%) patients in the Humira® arm reported at least one adverse event. The most prevalent adverse events were local (8.82% with CinnoRA®, 17.65% with Humira®) and respiratory (8.82% with CinnoRA®, 20.59% with Humira®) adverse effects (Table 3).

**Table 3:** Summary of information relating to the adverse effects in the treatment arms

Adverse effects (AE)		Number (%)	
Organ systems	Type	CinnoRA®	Humira®
Patients with at least one AE, total*		24 (35.29)	30 (44.12)
Dermatologic	Hives	2 (2.94)	5 (7.35)
	Swelling		
	Rash		
Local	Inject site react., erythema	6 (8.82)	12 (17.65)
	Inject site react., itching		
	Inject site react., hemorrhage		
	Inject site react., swelling		
	Inject site react., pain		
Respiratory	Sinusitis	6 (8.82)	14 (20.59)
	Flu-like syndrome		
	Difficulty breathing		
	Respiratory infection		
Gastrointestinal	Nausea	5 (7.35)	2 (2.94)
	Abdominal pain		
Central nervous system	Headache	4 (5.88)	4 (5.88)
Renal	Urinary tract infection	1 (1.47)	1 (1.47)
Neuromuscular	Back pain	1 (1.47)	2 (2.94)
Other	Other	11 (16.18)	6 (8.82)

*Data were analyzed using the Pearson chi-squared test (*p* value = 0.29). *react.* reaction

## Discussion

Randomized clinical trials demonstrating comparable efficacy, safety and tolerability of the biosimilar and innovator products are absolutely necessary as well as analytical evidence to establish similar physicochemical and biological actions of the products. Biosimilars improve the availability of more affordable products while offering similar efficacy and safety to the reference product [18, 25–27]. The perception of biosimilarity has been changed over recent years, especially in rheumatology. In September 2016, AMJEVITA™ (Amgen®, Thousand Oaks) was approved by the FDA, as a biosimilar adalimumab for treatment of seven inflammatory diseases. Similarly, Exemptia™, another adalimumab biosimilar, was approved in India. Etanercept and infliximab biosimilars were also authorized in various indications. In May 2016, Inflectra™ was approved by the European Medicines Agency (EMA) and the FDA as an infliximab biosimilar for all indications of the reference infliximab, including RA, AS, PsA, PsO, CD, and UC. In the case of etanercept, the biosimilar HD203 was recently approved in South Korea. In addition, Samsung Bioepis’s SB4, known as BRENZYS™ (Samsung Bioepis Co., Ltd., Korea) received regulatory approval from the Korean Ministry of Food and Drug Safety (MFDS), the European Commission (EC), and the Australian Therapeutic Goods Administration (TGA) to be used as a biosimilar alternative to etanercept [18, 28].

In this randomized, double-blind, parallel-group, non-inferiority study, the efficacy and safety of CinnoRA® were compared with those of adalimumab in the treatment of adult patients with active RA. Several studies have assessed the efficacy of 40 mg adalimumab administered subcutaneously every other week, in terms of ACR criteria and DAS-based EULAR response. In the study of Bombardieri et al., the efficacy of adalimumab was evaluated in RA patients for a 12-week period. By the end of week 12, about 60% and 33% of patients achieved ACR20 and ACR50 responses, respectively. Based on EULAR criteria, 76% of patients attained a moderate response and 23% attained a good response. In addition, 12% of patients reached clinical remission, achieving a DAS28 less than 2.6 [29, 30]. In another study, Huang et al. assessed the efficacy and safety of adalimumab in combination with MTX by administrating 40 mg adalimumab every other week for 12 weeks. The results of this multicenter, randomized, double-blind, placebo-controlled clinical trial indicated that 57% of patients achieved ACR20 and 32.2% of them achieved ACR50 responses [31]. In line with previous studies, we evaluated the percentage of patients with moderate-to-good DAS-based EULAR response, which was our primary outcome measure. Additionally, we compared the number of patients reaching ACR 20, ACR50, and ACR70 responses, along with ESR, CRP, and HAQ status. The percentage of our patients achieving moderate and good EULAR responses increased significantly in both the CinnoRA® and the Humira® arms, and the difference between the two arms was not statistically significant. Further, the percentage of patients reaching ACR20, ACR50, and ACR70 increased significantly during the 6-month period. In the retrospective study of Takeuchi et al. investigating the ability of adalimumab to reduce disease activity in 167 patients with RA, the mean DAS28-ESR score decreased from 5.3 ± 1.3 at baseline to 3.5 ± 1.5 at week 52 (*p* < 0.0001), which is consistent with our findings [32].

Furst et al. conducted a double-blind, placebo-controlled study and assigned patients into groups receiving either adalimumab 40 mg subcutaneously every other week or placebo. The study aimed to evaluate the efficacy of adalimumab when given with standard antirheumatic therapy over 24 weeks in patients with active RA, who were not adequately responding to such therapies. Similarly, a 24-week follow-up period was considered to evaluate the efficacy and safety of test-adalimumab in rheumatic patients [33].

Aletaha et al. chose the 3-month time point as a critical decision point in the treatment of patients with RA. It seems that patients who have significantly improved by 3 months are more likely to reach their treatment target by 6 months. In fact, achieving responses at 3 months is a good indicator of remission at 12 months, whereas patients with poor responses at 3 months will probably benefit from changing the treatment [34]. In agreement with previous studies, patients in our study who had noticeable improvements at 3 months also had better remission status. Patients in the CinnoRA® arm responded to treatment within a shorter period of time; however, this difference was not statistically significant. In the study of Burmester et al. in rheumatic patients receiving adalimumab for approximately 5 years, the mean HAQ-disability index score decreased in the first 6 months and then remained steady till the end of the study [35]. Similarly, in our study the mean HAQ score decreased significantly in both treatment arms and the difference between the groups was not statistically significant. In fact, as an important therapeutic goal, adalimumab improved social and physical functions in rheumatic patients.

In the safety analyses of the study of Takeuchi et al., the most frequently noted adverse events during one year of treatment with adalimumab were reactions at the drug administration site, with a frequency of 11.40/100 patient-years [32]. Safety was evaluated based on the adverse events reported by patients. All the adverse events were summarized according to the *Medical Dictionary for Regulatory Activities system organ class* (MedDRA SOC). We did not notice any significant difference in the incidence of injection site reactions as the most prevalent adverse events between treatment arms, and our findings were consistent with previous studies. Despite having a negative PPD test at the beginning of the study, one of the patients had a positive PPD test 8 weeks later that was probably due to close contact with a patient infected with TB close to the time of study enrollment.

## Conclusion

Based on our findings, CinnoRA®, as a biosimilar adalimumab, was shown to be non-inferior to Humira® in the treatment of adult patients with active RA.

## Abbreviations

6-MP: 6-Mercaptopurine
ACR: American College of Rheumatology
ADR: Adverse drug reaction
AS: Ankylosing spondylitis
BDMARDs: Biological disease-modifying antirheumatic drugs
CD: Crohn’s disease
CHF: Chronic heart failure
CHO: Chinese hamster ovary cells
CI: Confidence interval
CRP: C-reactive protein
CXR: Chest X-ray
DAS28: Disease activity score in 28 joints
EC: European Commission
EMA: European Medicines Agency
ESR: Erythrocyte sedimentation rate
EULAR: European League Against Rheumatism
FDA: Food and Drug Administration
GCP: Good clinical practice
HAQ: Health assessment questionnaire
HAQ DI: Health assessment questionnaire disability index
HS: Hidradenitis suppurativa
IgG1: Immunoglobulin-G1
ITT: Intention-to-treat
JIA: Juvenile idiopathic arthritis
MedDRA SOC: Medical Dictionary for Regulatory Activities system organ class
MFDS: Food and drug safety
MI: Myocardial infarction
MTX: Methotrexate
NSAIDs: Non-steroidal anti-inflammatory drugs
PP: Per-protocol
PPD: Purified protein derivative
PsA: Psoriatic arthritis
PsO: Psoriasis
RA: Rheumatoid arthritis
sDMARDs: Synthetic disease-modifying antirheumatic drugs
TB: Tuberculosis
TGA: Australia’s therapeutic goods administration
TNF-α: Tumor necrosis factor-α
UC: Ulcerative colitis
